# Efficacy of adding levofloxacin to gemcitabine and nanoparticle-albumin-binding paclitaxel combination therapy in patients with advanced pancreatic cancer: study protocol for a multicenter, randomized phase 2 trial (T-CORE2201)

**DOI:** 10.1186/s12885-024-11973-9

**Published:** 2024-02-24

**Authors:** Hiroo Imai, Yasuhiro Sakamoto, Shin Takahashi, Hiroyuki Shibata, Atsushi Sato, Kazunori Otsuka, Kenji Amagai, Masanobu Takahashi, Takuhiro Yamaguchi, Chikashi Ishioka

**Affiliations:** 1 Department of Medical Oncology, Tohoku University Hospital, Sendai City, Japan; 2 Department of Medical Oncology, Osaki Citizen Hospital, Osaki City, Japan; 3 Chemotherapeutic Center, Sendai Kousei Hospital, Sendai City, Japan; 4 Department of Clinical Oncology, Akita University Graduate School of Medicine, Akita City, Japan; 5 Department of Medical Oncology, Hirosaki University Graduate School of Medicine, Hirosaki City, Japan; 6 Department of Medical Oncology, Miyagi Cancer Center, Natori City, Japan; 7 Department of Gastroenterology and Medical Oncology, Ibaraki Prefectural Central Hospital, Kasama City, Japan; 8 Clinical Research, Innovation and Education Center, Tohoku University Hospital, Sendai City, Japan; 9 Department of Clinical Oncology, Tohoku University Graduate School of Medicine, Sendai City, Japan

**Keywords:** Pancreatic cancer, Multicenter randomized phase 2 study, Levofloxacin, Gemcitabine, Nanoparticle albumin binding paclitaxel

## Abstract

**Background:**

Advanced pancreatic cancer is one of the leading causes of cancer-related deaths. For patients with advanced pancreatic cancer, gemcitabine and nanoparticle albumin-binding paclitaxel (nabPTX) combination (GEM/nabPTX) therapy is one of the recommended first-line treatments. Several retrospective studies have suggested that the addition of levofloxacin improves the efficacy of GEM/nabPTX therapy in patients with advanced pancreatic cancer. This prospective study aims to evaluate whether the addition of antibiotics improves the treatment efficacy of GEM/nabPTX as a first-line chemotherapy in patients with advanced pancreatic cancer.

**Methods:**

This multicenter, prospective, randomized, phase 2 trial will included 140 patients. Patients with advanced pancreatic cancer will be randomized in a 1:1 ratio to either the GEM/nabPTX therapy group or the GEM/nabPTX plus levofloxacin group. The primary endpoint for the two groups is median progression-free survival time (mPFS) for the full analysis set (FAS). The secondary endpoints for the two groups are median overall survival (mOS), response rate (RR), disease control rate (DCR), and adverse event (AE) for the FAS and mPFS, mOS, RR, DCR, and AE for the per-protocol set. This study will enroll patients treated with GEM/nabPTX as the first-line chemotherapy for stage IV pancreatic adenocarcinoma.

**Discussion:**

GEM/nabPTX is a standard first-line chemotherapy regimen for patients with advanced pancreatic cancer. Recently, the superiority of 5-fluorouracil, liposomal irinotecan, and oxaliplatin combination therapy (NALIRIFOX) to GEM/nabPTX as first-line therapy for pancreatic cancer has been reported. However, the efficacy of NALIRIFOX is inadequate. Based on previous retrospective studies, it is hypothesized that treatment efficacy will improve when levofloxacin is added to GEM/nabPTX therapy. If the AEs (such as leukopenia, neutropenia, and peripheral neuropathy) that occur at an increased rate with levofloxacin and GEM/nabPTX combination therapy can be carefully monitored and properly managed, this simple intervention can be expected to improve the prognosis of patients with advanced pancreatic cancer.

**Trial registration:**

This study was registered with the Japan Registry of Clinical Trials (jRCT; registry number: jRCTs021230005).

**Supplementary Information:**

The online version contains supplementary material available at 10.1186/s12885-024-11973-9.

## Background

Advanced pancreatic cancer is one of the leading causes of cancer-related death [1]. The 5-year survival rate of advanced pancreatic cancer is less than 5% [2]. Patients with advanced pancreatic cancer are typically treated with systemic chemotherapy. The National Comprehensive Cancer Network guideline suggests the use of 5-fluorouracil plus oxaliplatin plus irinotecan combination therapy (FOLFIRINOX) or gemcitabine plus nanoparticle albumin-binding paclitaxel (nabPTX) combination therapy (GEM/nabPTX) as first-line chemotherapy in patients with advanced pancreatic cancer [3].

The reported response rate, median progression-free survival time (mPFS), and median overall survival (mOS) time in patients with advanced pancreatic cancer are 31.6%, 6.4 months, and 11.1 months, respectively, with FOLFIRINOX therapy and 23.0%, 5.5 months, and 8.5 months, respectively, with GEM/nabPTX therapy [4, 5]. However, the treatment efficacy of these chemotherapy regimens remains inadequate.

Recently, an interaction was reported between the treatment efficacy of GEM and the use of antibiotics in vitro [6]. The study found that GEM is metabolized into the inactive form 2′,2′-difluorodeoxyuridine by the cytidine deaminase long isoform (CDD_L_) secreted by intratumoral bacteria. In vivo analysis showed that treatment with antibiotics reduced the numbers of bacteria in the xenograft tumor tissue and improved the efficacy of GEM. Previous studies have also shown that bacteria secreting the CDD_L_ infiltrate the pancreatic cancer tissues in more than 70% of patients with pancreatic cancer [6, 7]. CDD_L_ is secreted by several types of bacteria including the *Citrobacter*, *Enterobacter*, or *Klebsiella* [8]. These bacterial genera are sensitive to quinolone antibiotics [9]. Based on these findings, it has been hypothesized that the concomitant administration of quinolone antibiotics in patients with pancreatic cancer improves the efficacy of GEM by reducing the number of CDD_L_- secreting bacteria in the tumor tissue.

A retrospective study reported that the addition of antibiotics improved the efficacy of a GEM-containing regimen in patients with advanced malignant tumors, including those with pancreatic cancer [10]. In that study, the mPFS and mOS in the antibiotic untreated group or the antibiotic-treated group were 2.5 (95% confidence interval [CI]: 1.86–3.73) or 4.9 (95% CI: 3.47–6.0) months (*p* < 0.0001) and 7.53 (95% CI: 5.63–9.57) months or 13.83 (95% CI: 10.83–16.43) months (*p* < 0.001), respectively. Further retrospective studies have reported an improvement in the efficacy of GEM-containing regimens in patients with pancreatic cancer through the addition of antibiotics [11, 12].

In this multicenter randomized phase 2 study, we aim to evaluate the treatment efficacy and safety of GEM/nabPTX and levofloxacin combination therapy as a first-line treatment in patients with advanced pancreatic cancer.

### Trial design

**Fig. 1:**
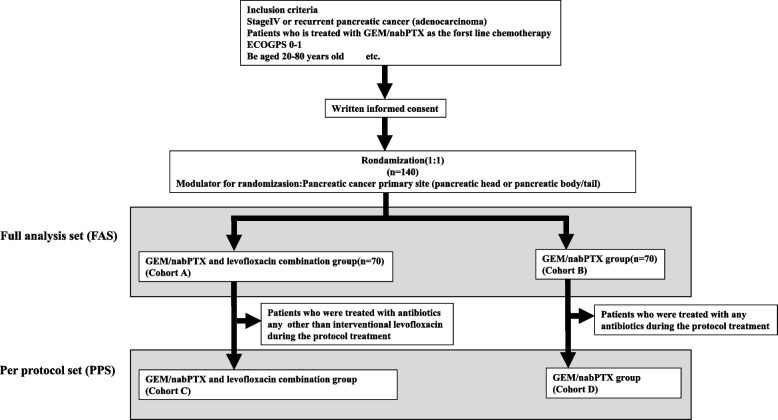
Study schema

In this multicenter, prospective, randomized, phase 2 trial, patients with advanced pancreatic cancer will be randomized in a 1:1 ratio to either the GEM/nabPTX therapy group or the GEM/nabPTX plus levofloxacin group. A total of 140 patients will be enrolled, with each group comprising 70 patients. The study design is illustrated in Fig. 1.

The following seven institutions will participate in the study: Tohoku University Hospital; the Department of Medical Oncology, Osaki Citizen Hospital; Sendai Kousei Hospital; Akita University Graduate School of Medicine; Hirosaki University Graduate School of Medicine; Miyagi Cancer Center; and Ibaraki Prefectural Central Hospital.

The study period is from May 19, 2023 (date of publication of the Japan Registry of Clinical Trials (jRCT)) to November 31, 2027. The participants will be followed up for 2 years.

### Participants

The inclusion and exclusion criteria are summarized below. The complete lists of inclusion and exclusion criteria are described in Supplementary Table 1.

### Inclusion criteria

The inclusion criteria are as follows: an Eastern Cooperative Oncology Group performance status (ECOG-PS) of 0 or 1, age > 20 and < 80 years, stage IV pancreatic adenocarcinoma treated with GEM/nabPTX as first-line treatment, the presence of at least one RECIST version 1.1 evaluable lesion, and patients eligible for protocol treatment and oral levofloxacin based on blood test results.

### Exclusion criteria

The exclusion criteria are as follows: GEM or nabPTX treatment within 6 months prior to enrollment or focal radiation, a history of other treatments that may affect the efficacy of the protocol treatment, inability to take oral levofloxacin or antibiotic treatment for more than five consecutive days within 1 month prior to enrollment, treatment with drugs contraindicated for use with levofloxacin, other conditions that may affect the efficacy or safety of levofloxacin, and inability to continue the protocol treatment.

### Enrollment and allocation

Eligible patients will be randomized without blinding to the GEM/nabPTX or GEM/nabPTX plus levofloxacin combination groups. Randomization will be performed using the Viedoc 4™ (Electric Data Capture (EDC) system) by the Clinical Research, Innovation and Education Center, Tohoku University Hospital (CRIETO).

### Interventions

The dose and schedule of the GEM/nabPTX therapy (the same for each group in this study) are GEM 1,000 mg/m^2^ and nabPTX 125 mg/m^2^ on days 1, 8, and 15 every 4 weeks.

Patients in the GEM/nabPTX plus levofloxacin group will receive levofloxacin in addition to GEM/nabPTX therapy.

**Fig. 2:**
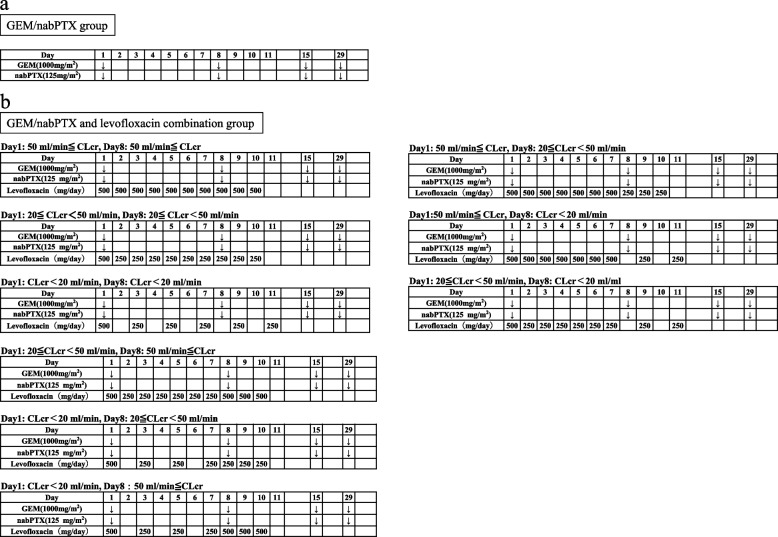
The schedule of the protocol treatment of the present study. The schedule of GEM/nabPTX group is shown in **a**, and of GEM/nabPTX and levofloxacin combination group is shown in **b**

The dose and schedule of GEM/nabPTX and levofloxacin therapy are shown in Fig. 2a and b, respectively. Almost all of levofloxacin is metabolized by the kidneys and excreted from the body. Therefore, dosage should be adjusted according to renal function. In this clinical trial, the dose of levofloxacin will be adjusted based on the creatinine clearance calculated by the Cockcroft-Gault formula using blood sampling data from day 1 and day 8. Doses have been determined based on previous reports.

As shown in Fig. 2b, levofloxacin was adjusted according to the renal function status (based on previous reports [13]) and administered for 10 or 11 days from the initiation of GEM/nabPTX therapy. The duration of oral levofloxacin therapy has been extensively considered to minimize the occurrence of adverse events and to maintain patient medication compliance. Patients will return the unused levofloxacin and an empty package. The participant timelines are presented in Table 1. Consent will be obtained from patients who meet the inclusion criteria at the pre-enrollment visit, and protocol treatment will be initiated. ECOG-PS, blood pressure, heart rate, body temperature, and adverse events will be monitored at each outpatient visit. Imaging studies will be performed every 2 months (± 1 week). At the end of the protocol treatment, height, weight, blood sampling data, and adverse events will be monitored, and tumor marker testing and imaging studies will be performed. After completion of the protocol treatment, imaging studies will be performed every 2 months (± 1 week) and survival survey will continue.

**Table 1:** Participant time line

				**Duration of protocol treatment**									**Termination or discontinuition of protocol**	**Follow up**
				**Course 1**				**Course 2-**				**2 months ∓ 1 week**		
	**Screening**	**Consent**	**Allocation**	**Day1**	**Day8**	**Day15**	**Day22**	**Day1**	**Day8**	**Day15**	**Day22**			
**Registration/allocation**														
**Imformed concent/tenporary registration**		**X**												
**Official registration**			**X**											
**Allocation**			**X**											
**Protocol treatment**														
**GEM**				**X**	**X**	**X**		**X**	**X**	**X**				
**nabPTX**				**X**	**X**	**X**		**X**	**X**	**X**				
**levofloxacin**				**Day1-10(11) administration**										
**Safety evaluation items**														
**Background**	**X**	**X**												
**Height**	**X**													
**Body weight**	**X**			**X**				**X**					**X**	
**ECOG PS**	**X**			**X**	**X**	**X**		**X**	**X**	**X**			**X**	
**Blood pressure**	**X**			**X**	**X**	**X**		**X**	**X**	**X**				
**Heart rate**	**X**			**X**	**X**	**X**		**X**	**X**	**X**				
**Body temprature**	**X**			**X**	**X**	**X**		**X**	**X**	**X**				
**Full blood count**	**X**			**X**	**X**	**X**		**X**	**X**	**X**			**X**	
**Serum biochemistry**	**X**			**X**	**X**	**X**		**X**	**X**	**X**			**X**	
**Urinalysis**	**X**													
**Adverse events**				**X**	**X**	**X**		**X**	**X**	**X**			**X**	
**Concomitant medication**	**X**													
**Hepatisis B/C related tests**	**X**													
**Pregnancy test**	**X**													
**Efficacy evaluation items**														
**Tumor marker**	**X**			**X**				**X**					**X**	
**CT/MRI(Imaging test)**	**X**											**X**	**X**	**X**
**Survival survey**													**X**	**X**

Administration of granulocyte-colony stimulating factor (G-CSF) to increase neutrophil count is allowed for neutropenia, as is the administration of medications such as 5-HT_3_ antagonists, aprepitant, corticosteroids, metoclopramide, and prochlorperazine for GEM/nabPTX-induced nausea. Radiation for pain relief is allowed for pain caused by bone metastases (the radiation site and dose are determined in consultation with the radiologist). Administration of medications for other complications (e.g., opioids, bisphosphonates, anti-RNKL antibodies.) is allowed. The use of antibiotics (any type) is allowed for the treatment of any infection in both groups at the discretion of the treating physician. For grade 3 or higher neutropenia, treatment with antibiotics (any type) is allowed at the discretion of the treating physician.

The prohibited therapies included treatment with any anticancer drug other than GEM or nabPTX, irradiation other than palliative irradiation, surgery to resect the primary or metastatic lesion, tumor embolization, or any biological response modifiers.

### Withdrawal criteria

#### GEM/nabPTX therapy

GEM/nabPTX therapy in each group will be repeated until the following criteria for withdrawal are met: 1) exacerbation of pancreatic cancer (exacerbations as defined by RECIST version 1.1 and clinical progression such as worsening of non-target lesions (ascites or pleural effusion), worsening of subjective symptoms, or decrease in ECOG-PS) after starting the protocol treatment; 2) discontinuation of the protocol treatment because of the occurrence of adverse events; 3) patient request for discontinuation of the protocol treatment because of the occurrence of adverse events; 4) death during the protocol treatment; 5) delay in the start date of the next term of the protocol treatment by more than 28 days from the date of the previous protocol treatment, due to unavoidable circumstances other than the occurrence of adverse events; 6) ineligibility to participate in the study determined after enrollment or a decision by a physician to discontinue the protocol treatment for any reason.

#### Levofloxacin

Immediate discontinuation of levofloxacin is required if any of the following occur:

1. Any type and grade of allergy or anaphylaxis following the use of levofloxacin.
2. Liver dysfunction of grade 3 or higher. Levofloxacin administration will not be resumed even if liver function improves after discontinuation of the antibiotic. The actual dose of levofloxacin will be monitored in each patient and the correlation between the dose of levofloxacin and the efficacy of the protocol treatment will be analyzed.

#### Subsequent treatment

Subsequent-treatment after the completion of the protocol treatment is not specified.

### Statistical analyses

The full analysis set (FAS; Fig. 1) is defined as patients who meet the inclusion criteria and are treated using the protocol at least once. The per-protocol set (PPS, Fig. 1) is defined as patients subtracted from the FAS because of treatment with antibiotics other than levofloxacin (interventional treatment) during the protocol treatment. All data will be statistically analyzed at the Clinical Research, Innovation, and Education Center, Tohoku University Hospital (CRIETO).

### Definition of sample size

Based on three previous retrospective studies [10–12], the mPFS duration is 3 months for the GEM/nabPTX therapy group and 5 months for the GEM/nabPTX plus levofloxacin group. The required number of patients has been calculated based on an enrollment period of 3 years, a follow-up period of 1 year, a two-sided significance level of 5%, and a statistical power of 85%. According to the calculations, 64 cases in each group are required to achieve a significant difference. Since a dropout rate of 10% is expected in each group, 64 + 64 × 0.1 = 70 cases (70 cases in both groups × 2 = 140 cases) is the number of cases needed to obtain a significant difference in this clinical trial.

### Outcomes

Using the FAS (cohorts A and B (Fig. 1)), the mPFS will be compared between cohorts A and B as the primary endpoint. The secondary endpoints will be the median overall survival (mOS), response rate (RR), disease control rate (DCR), and adverse events (AEs) of the FAS. The mPFS, mOS, RR, DCR, and AE of the PPS (cohorts C and D) will also be evaluated as secondary endpoints. The response rate will be calculated in patients with at least two imaging assessments to determine efficacy, and the response rate will be compared between the two groups. DCR is defined as the time from the start of the protocol treatment to the time when pancreatic cancer is observed. It is calculated as: the (number of patients with CR + PR + SD) / (number of patients with CR + PR + SD + PD). Monitoring will be performed for all Grade 3 or higher adverse events (including AEs of special interest with concomitant antibiotics and chemotherapy) observed during the study period.

The principal investigator, co-investigators, and research staff will enter the clinical information into the EDC system (Viedoc 4™) as soon as the clinical data of each participant are available. This prevents the leakage of personal information; investigators will use the participant identification number when entering the information into the EDC system (Viedoc 4™). Data monitoring will be performed by the data center at CRIETO.

AEs will be graded according to the Common Terminology Criteria for Adverse Events version 5.0. All AEs (> grade 3) in this trial will be entered into the EDC system. If severe AEs (SAEs) are observed in any patient in this trial, the SAE report from each responsible physician will be e-mailed or faxed to the T-CORE study authorities within 72 h of the event and reported by the T-CORE authorities to the principal investigator (Chikashi Ishioka).

### Exploratory study

Stool, blood, and cancer tissue samples will be collected from patients participating in this trial and agreeing to provide the required samples. Stool and blood samples will be collected on days 1 and 8. We hypothesize that not only changes in the amount of bacteria in tissues, but also changes in metabolites in serum and feces based on changes in the bacterial flora produced in the body by the antibiotic treatment may be involved in improving the efficacy of GEM/nabPTX. Mass spectrometry will be used to analyze changes in metabolites in serum and feces due to antibiotic treatment. Since metabolites with altered levels are likely to affect the efficacy of GEM/nabPTX, we will focus on these metabolites and conduct basic experiments.

## Discussion

GEM/nabPTX therapy is a standard first-line chemotherapy for patients with advanced pancreatic cancer [5]. A previous study reported a median PFS of 5.5 months with GEM/nabPTX therapy. The NAPOLI-3 trial comparing 5-fluorouracil, liposomal irinotecan, and oxaliplatin combination therapy (NALIRIFOX) to GEM/nabPTX as a first-line therapy for pancreatic cancer showed the superiority of NALIRIFOX over GEM/nabPTX in OS and PFS [14]. However, the previously reported treatment efficacy of first-line chemotherapy for advanced pancreatic cancer is low. Therefore, improving the treatment efficacy of currently available chemotherapies for patients with advanced pancreatic cancer remains an important challenge.

Pancreatic cancer is associated with a wide variety of gene mutations [15] and the pancreatic cancer tissue microenvironment is composed of cancer cells, cancer-associated fibroblasts, and immune cells [16]. Various subtypes of pancreatic cancer based on these characteristics have emerged. Changes in gut microbiota have been reported to affect the efficacy of cytotoxic anticancer drugs [17]. Furthermore, metabolites secreted from gut microbiota have been reported to affect various cancers via blood [18]. As changes in gut bacteria induced by antimicrobial agents may alter the types and amounts of metabolites in stool and blood, in this study, we will conduct translational research using blood and stool samples, as described in the Exploratory Research section, to identify metabolites involved in the therapeutic effect of GEM/nabPTX in pancreatic cancer. The discovery of these metabolites may lead to the development of new therapeutic targets to improve the therapeutic efficacy of GEM/nabPTX in pancreatic cancer.

Quinolones are broad-spectrum antibiotics. Quinolones are generally safe and are commonly used for pneumonia and other infections. Despite their safety, an increase in the risk of AEs such as tendinopathy, ruptured aneurysms, aortic dissection, and peripheral neuropathy, has been reported although less frequently [19]. Gemcitabine and nabPTX, which are used as the protocol treatment in this trial, may cause peripheral neuropathy; therefore, patients in the levofloxacin combination arm in this trial will be monitored closely for exacerbations of peripheral neuropathy.

Several retrospective studies have reported that antibiotic treatment improves the therapeutic efficacy of GEM in patients with pancreatic cancer [8, 10–12]. Based on these results, the treatment efficacy of GEM may improve in the levofloxacin-treated group. In the future, we intend to conduct a Phase 3 clinical trial to evaluate the superiority of GEM/nabPTX and levofloxacin combination therapy over GEM/nabPTX therapy alone. If the addition of levofloxacin to GEM/nabPTX therapy improves the treatment efficacy of GEM/nabPTX therapy in future clinical trials, it can be used for improving the therapeutic outcomes of patients with advanced pancreatic cancer. However, in a previous retrospective analysis, the incidence of leukopenia and neutropenia in gemcitabine-containing regimens was increased in patients treated with the levofloxacin combination [10]. The possibility of an increased incidence of leukopenia and neutropenia in the levofloxacin group will also be carefully monitored in this study. For sample size calculation, both PFS for the GEM/nabPTX group and for the GEM/nabPTX plus antibiotics group were required. A previous prospective study showed that the PFS of GEM/nabPTX therapy was 5.5 months, but this study did not include data to estimate the PFS with the addition of antibiotics [5]. Therefore, the data from the previous prospective study cannot be used to calculate the sample size in this study. And the sample size of this study was determined based on the data from previous retrospective analyses [10–12]. If the results of this study indicate that the addition of levofloxacin improves the therapeutic effect of GEM/nabPTX, this combined treatment may help to improve the prognosis of patients with advanced pancreatic cancer in the future.

### Supplementary Information

**Additional file 1: Supplemental Table 1. **Inclusion and exclusion criteria.

## Data Availability

The datasets used and/or analyzed in the current study are available from the corresponding author upon reasonable request.

## Abbreviations

AEs: Adverse events
CDDL: Cytidine deaminase long isoform
CI: Confidence interval
DCR: Disease control rate
EDC: Electric Data Capture
FAS: Full analysis set
FOLFIRINOX: 5-Fluorouracil plus oxaliplatin plus irinotecan
GEM/nabPTX: Gemcitabine and nanoparticle albumin-binding paclitaxel
HRR: Homologous recombination repair
jRCT: Japan Registry of Clinical Trials
mOS: Median overall survival
mPFS: Median progression-free survival time
nabPTX: Nanoparticle albumin-binding paclitaxel
PPS: Per-protocol set
RR: Response rate
